# Low FODMAP Diet and Probiotics in Irritable Bowel Syndrome: A Systematic Review With Network Meta-analysis

**DOI:** 10.3389/fphar.2022.853011

**Published:** 2022-03-09

**Authors:** Chao-Rong Xie, Bin Tang, Yun-Zhou Shi, Wen-Yan Peng, Kun Ye, Qing-Feng Tao, Shu-Guang Yu, Hui Zheng, Min Chen

**Affiliations:** ^1^ The Third Hospital/Acupuncture and Tuina School, Chengdu University of Traditional Chinese Medicine, Chengdu, China; ^2^ Digestive Department, People’s Hospital of Zhongjiang County, Zhongjiang, China; ^3^ Department of Colorectal Diseases, Hospital of Chengdu University of Traditional Chinese Medicine, Chengdu University of Traditional Chinese Medicine, Chengdu, China

**Keywords:** irritable bowel syndrome, probiotics, low fermentable oligosaccharides, disaccharides, monosaccharides, and polyols, component network meta-analysis, systematic review

## Abstract

**Background:** Probiotic and low fermentable oligosaccharide, disaccharide, monosaccharide, and polyol (FODMAP) diet are two commonly used management approaches for patients with irritable bowel syndrome (IBS). We aimed to evaluate the most effective combinations and components among different probiotics or low FODMAP diet through component network meta-analysis (NMA).

**Methods:** We searched Embase, Ovid Medline, and Web of Science from inception to 21 January 2021. Randomized controlled trials (RCTs) examining the efficacy of probiotics and low FODMAP diet for IBS were included, with placebo, sham diet, or conventional treatments as controls. Binary outcomes were compared among treatments using the relative ratio (RR). A minimally contextualized framework recommended by the GRADE group was used to evaluate the certainty of evidence. The primary efficacy outcome was the relief of global IBS symptoms, and the secondary efficacy outcome was the reduction in IBS symptom scores or abdominal pain scores.

**Key Results:** We included 76 RCTs (n = 8058) after screening 1940 articles. Eight RCTs were classified as low risk of bias. Standard network meta-analysis (NMA) showed that *Lactobacillus* (RR 1.74, 95% CI 1.22–2.48) and *Bifidobacterium* (RR 1.76, 95% CI 1.01–3.07) were the most effective for the primary efficacy outcome (high certainty evidence); component NMA showed that *Bacillus* (RR 5.67, 95% CI 1.88 to 17.08, *p* = 0.002) and *Lactobacillus* (RR 1.42, 95% CI 1.07 to 1.91, *p* = 0.017) were among the most effective components. The results of standard NMA and CNMA analysis of the improvement of overall IBS symptom scores or abdominal pain scores were consistent with this finding.

**Conclusion:** *Lactobacillus* was the most effective component for the relief of IBS symptoms; *Bifidobacterium* and *Bacillus* were possibly effective and need further verification.

**Systematic Review Registration:** website, identifier registration number.

## Introduction

Irritable bowel syndrome (IBS) is a chronic, often debilitating bowel disease because of the disorder of the brain–gut axis (Drossman and Hasler, 2016; Ford et al., 2017). IBS is characterized by recurrent abdominal pain that is correlated with changes in stool consistency or frequency (Lacy et al., 2016; Ford et al., 2020). IBS has a substantial impact on the quality of life and social functioning (Buono et al., 2017; Frändemark et al., 2018), and affects 5–10% of the general population (Sperber et al., 2021).

Management therapies with diet and probiotics were of great interest in patients with IBS since they are safe and well-tolerated. The mainstream dietary management includes dietary fiber, with low fermentable oligosaccharide, disaccharide, monosaccharide, and polyol (FODMAP) diet, which is a gluten-free diet (Ford et al., 2020). There is little evidence supporting the use of a gluten-free diet in IBS, and it is still ambiguous whether patients should increase their dietary fiber intake to mitigate IBS symptoms (Dionne et al., 2018). On the contrary, low FODMAP diet and probiotics were shown to be effective for IBS in several systematic reviews (Li et al., 2020; Niu and Xiao, 2020; Black et al., 2021; van Lanen et al., 2021). In the 2020 ACG guidelines (Lacy et al., 2021), probiotics and a low FODMAP diet are recommended to alleviate IBS symptoms before escalating to medical therapies or as adjuncts to medical therapies.

Although numerous studies have shown that probiotics are effective in the treatment of IBS, whether particular combinations, species, or strains of probiotics are more effective than the others remains unclear (Ford et al., 2018). A network meta-analysis (NMA) showed that different probiotics had different responder rates, and a combination of *Lactobacillus* and *Bifidobacterium* might have a better treatment effect on IBS. Owing to the small number of included studies and the method of analysis applied, the NMA could not reach a firm conclusion. One recent study demonstrated that a combination of both probiotics and a low FODMAP diet enlarged the treatment effect (Staudacher et al., 2017), but it is unclear which probiotic strains are more effective and which components contribute more than the others. Component NMA has been developed to identify the most effective component of complex intervention combinations. Therefore, we conducted a systematic review on component NMA, aiming to study the comparative effectiveness of differential probiotics, low FODMAP diet, and their combinations in the management of IBS, and to identify the most effective components.

## Methods

### Data Source

We searched Embase, Ovid Medline, and Web of Science from inception to 21 January 2021, for RCTs testing the efficacy of probiotics or a low FODMAP diet in the management of IBS. A supplementary search was performed on 21 January 2022, and 4 trials were added. A search strategy for the databases is provided in Supplementary Table S1. We read the references of relevant reviews and the retrieved studies, searching for any missing trials.

### Study Selection

RCTs meeting the following criteria were included: participants were diagnosed with IBS based on either a clinician’s opinion, or any of the following diagnostic criteria^16^—a Manning, Kruis score, Rome I, II, III, or IV; assessing the efficacy of probiotics or low FODMAP diet in IBS treatment by comparing with active control, placebo, sham diet, or high FODMAP diet; with at least one targeted outcome measurement—relief of IBS symptoms, overall IBS symptom scores or abdominal pain scores, or adverse events. RCTs with any of the following conditions were excluded: crossover design and data not reported by stages, details of the accompanying treatments unrevealed, and full-text copy unavailable.

Two reviewers (CRX and KY) independently screened possible candidates by reading titles and abstracts. Full-text copies of potentially eligible RCTs were acquired for further evaluation. The discrepancy in the inclusion of an RCT was solved by group discussion and arbitrated by a reviewer (HZ).

### Data Extraction and Risk of Bias Assessment

Two reviewers (WYP and QFT) obtained the necessary information from eligible RCTs. Data extractions included the following: 1) trial characteristics like name of the first author, publication year, country, study type, and sample size; 2) participant characteristics like diagnostic criteria, IBS subtype, mean age, and proportion of females; 3) intervention and control: name of the intervention or control, dosage, and frequency of treatment, duration of treatment, and follow-up time; and 4) outcome measures: name of the outcome, the number of participants allocated to the intervention or control, parameters like mean standard deviation, and the number of events.

The primary efficacy outcome was the relief of global IBS symptoms at the end of treatment, which was determined by a question of whether adequate relief of IBS symptoms was achieved or a reduction of at least 50 points in the IBS symptom severity score (IBS-SSS) (Francis et al., 1997; Irvine et al., 2016).

The secondary efficacy outcome was the reduction in IBS symptom scores or abdominal pain scores, which were measured by differential scales and were preferentially selected in the following order: the IBS-SSS scale, 100-mm visual analog scale (VAS), 11-point numerical rating scale (NRS), subjects’ global assessment (SGA) scale, gastrointestinal symptom rating scale (GSRS), and other scoring systems.

The risk of bias (RoB) of the included trials was evaluated using the Cochrane RoB tool (Sterne et al., 2019). We judged a trial with a low RoB when all the five domains (randomization process, deviations from intended interventions, missing outcome data, measurement of the outcome, and selection of the reported result) were classified as low RoB. The certainty of the evidence was evaluated by using the Grading of Recommendations Assessment, Development, and Evaluation (GRADE) approach that was specifically developed for concluding a network meta-analysis, and the GRADE approach adopted a minimally contextualized framework that was described elsewhere in detail (Brignardello–Petersen et al., 2020). The certainty of the evidence was expressed as high certainty (moderate to high certainty evidence) and low certainty (low to very low certainty evidence). The classification of intervention was expressed as category 2 (among the most effective), category 1 (inferior to the most effective, or superior to the least effective), and category 0 (among the least effective) (Brignardello–Petersen et al., 2020). Trained GRADE methodologists analyzed the data to assess the quality of evidence, given the strength of recommendation.

### Data Synthesis

We performed standard network meta-analysis (NMA) comparing the comparative effectiveness of different treatments or treatment combinations through a frequentist approach based on the electrical networks and graph theory (Rücker, 2012). Placebo was used as a reference comparator to calculate the effect size of the treatment. The effect size of binary variables was calculated as the relative ratio (RR); RR and its 95% confidence interval (95% CI) were presented, and a 95% CI containing the null value (RR = 1) indicated the insignificant difference between a treatment and placebo. The effect size of continuous variables was calculated as standardized mean difference (SMD); SMD and its 95% confidence interval (95% CI) were presented, and a 95% CI containing the null value (SMD = 0) indicated the insignificant difference between a treatment and placebo. Two forest plots summarizing both direct and indirect evidence of a treatment’s RR and SMD were presented. The treatments were ranked by the surface under the cumulative ranking (SUCRA) score—a measurement parameter that evaluates which treatment is the most effective one.

Component NMA was performed by using an additive component NMA model (Rücker et al., 2020), which hypothesizes that the effect size of complex interventions with multiple treatment components is the sum of the effect of the components. To identify the most effective component, the effect size of each component was calculated concerning placebo.

The approach of calculating RR and SMD is the same as the standard NMA, and the Z test was used to measure whether there was any significant inconsistency between them with a cutoff point of *p* < 0.05.

Heterogeneity of the NMA was examined by using the global *I* ^*2*^ statistics, in which an *I* ^*2*^ value less than 40% was considered as unimportant heterogeneity, as stated in the Cochrane handbook 5.1. A design-by-treatment analysis was performed to find out the source of heterogeneity, and a sensitivity analysis was subsequently performed to check the robustness of the findings by excluding the RCTs that caused significant heterogeneity. We examined the consistency of the NMA by comparing the direct and indirect comparison estimates.

## Results

### Characteristics of the Included RCTs

**FIGURE 1:**
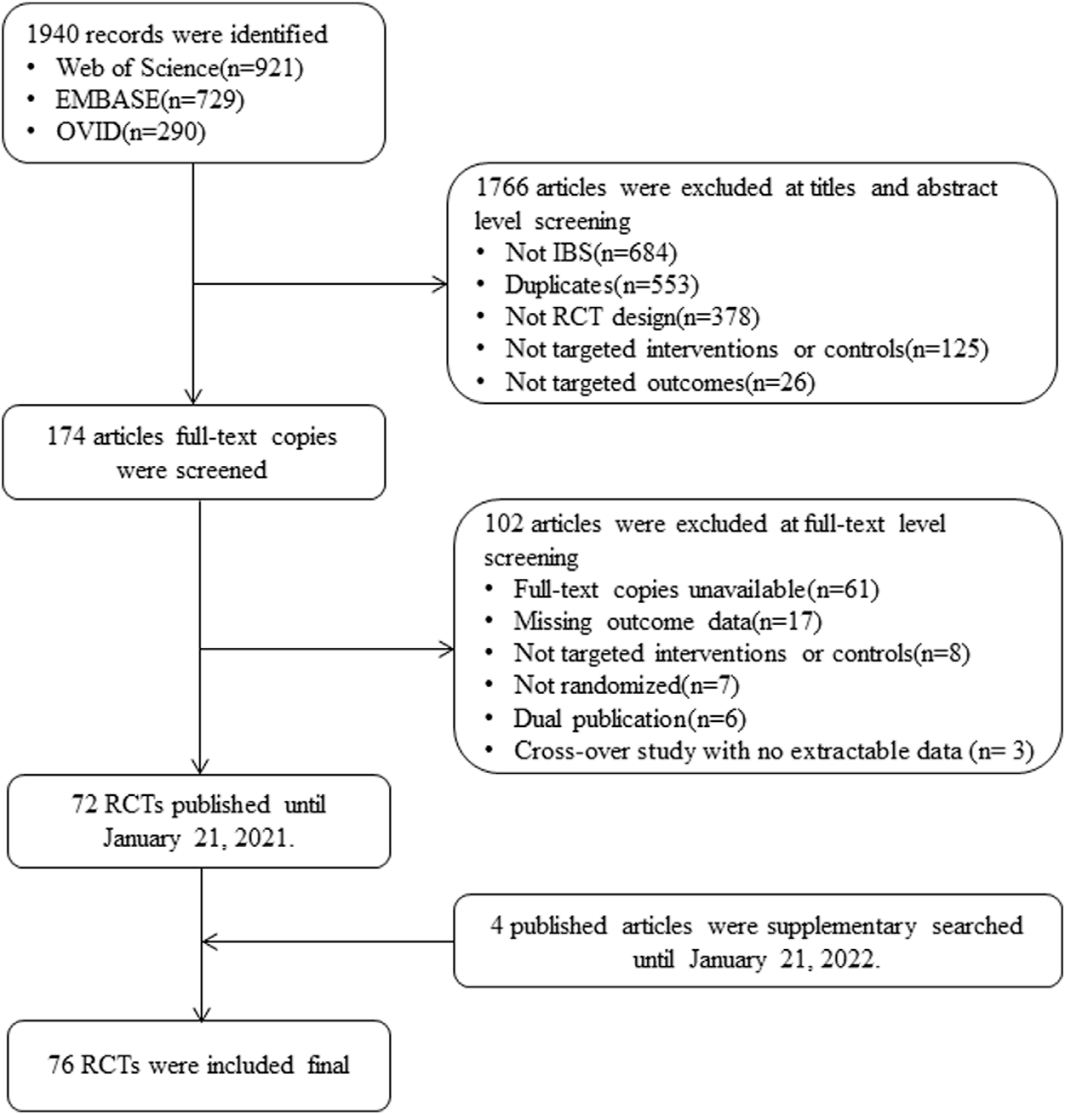
Study flowchart. Abbreviations: IBS, irritable bowel syndrome; RCT, randomized controlled trial.

We included 76 RCTs (n = 8058) after screening 1940 possible candidates; Figure 1 shows the detailed screening process. The included RCTs were conducted in 26 countries, with the sample sizes ranging from 19 to 443 participants (33–123 participants in the RCTs of low FODMAP diet group and 19 to 443 participants in the RCTs of the probiotics group). Among the subtypes of IBS, 17 (22.4%) trials were IBS-D, 3 (3.9%) were IBS-C, and the remaining 56 (73.7%) were a mixture of multiple subtypes.

The mean age of the overall population ranged from 11.5 to 59.3 years (11.5–51 years in the low FODMAP diet group and 21.8–59.3 years in the probiotics group). The proportion of women in the RCTs ranged from 26.4 to 100% (58–100% in the low FODMAP diet group and 26.4–100% in the probiotics group). The study duration ranged from 2 to 48 weeks (2–16 weeks in the low FODMAP diet group and 4–48 weeks in the probiotics group); there were 63 (82.9%) RCTs ranging from 4 to 12 weeks, and 44 (57.9%) RCTs ranging from 4 to 8 weeks. Thirty-one trials assessed a combination of probiotics (Kim et al., 2003; Kim et al., 2005; Kajander et al., 2005; Kim et al., 2006; Guyonnet et al., 2007; Zeng et al., 2008; Drouault–Holowacz et al., 2008; Kajander et al., 2008; Williams et al., 2009; Hong et al., 2009; Simren et al., 2010; Sondergaard et al., 2011; Ringel-Kulka et al., 2011; Michail and Kenche, 2011; Cha et al., 2012; Cui and Hu, 2012; Begtrup et al., 2013; Ko et al., 2013; Roberts et al., 2013; Jafari et al., 2014; Yoon et al., 2014; Ludidi et al., 2014; Lorenzo–Zuniga et al., 2014; Sisson et al., 2014; Yoon et al., 2015; Wong et al., 2015; Staudacher et al., 2017; Hod et al., 2017; Kim et al., 2020; Skrzydło–Radomańska et al., 2021; Barraza–Ortiz et al., 2021), seventeen trials assessed *Lactobacillus* (Nobaek et al., 2000; Niedzielin et al., 2001; Niv et al., 2005; Sinn et al., 2008; Farup et al., 2012; Ducrotte et al., 2012; Dapoigny et al., 2012; Murakami et al., 2012; Stevenson et al., 2014; Pedersen et al., 2014; Thijssen et al., 2016; Lyra et al., 2016; Shin et al., 2018; Oh et al., 2019; Sadrin et al., 2020; Lewis et al., 2020; Martoni et al., 2020), eleven trials assessed the effect of a low FODMAP diet (Staudacher et al., 2012; Pedersen et al., 2014; Böhn et al., 2015; Chumpitazi et al., 2015; Eswaran et al., 2016; McIntosh et al., 2017; Staudacher et al., 2017; Patcharatrakul et al., 2019; Darvishmoghadam et al., 2019; Wilson et al., 2020; Goyal et al., 2021), eight trials assessed *Bifidobacterium* (Whorwell et al., 2006; Agrawal et al., 2009; Guglielmetti et al., 2011; Charbonneau et al., 2013; Pinto–Sanchez et al., 2017; Andresen et al., 2020; Lewis et al., 2020; Martoni et al., 2020), and five trials assessed *Saccharomyces* (Choi et al., 2011; Abbas et al., 2014; Pineton de Chambrun et al., 2015; Spiller et al., 2016; Gayathri et al., 2020). Supplementary Table S2 shows detailed characteristics of the included RCTs.

9 (11.8%) of the RCTs were classified with overall low RoB, 67 (88.2%) of the RCTs were classified with some concerns. The details were as follows: 37 (48.7%) of the RCTs were classified with low RoB in the randomization process, 51 (67.1%) were classified with low RoB in deviations from intended interventions, 64 (84.2%) were classified with low RoB in missing outcome data, 31 (40.8%) were classified with low RoB in the measurement of the outcome, and all the RCTs were with low RoB in the selection of the reported result. The RoB assessment of individual RCTs is shown in Supplementary Figure S1.

### Relief of Global IBS Symptoms

**FIGURE 2:**
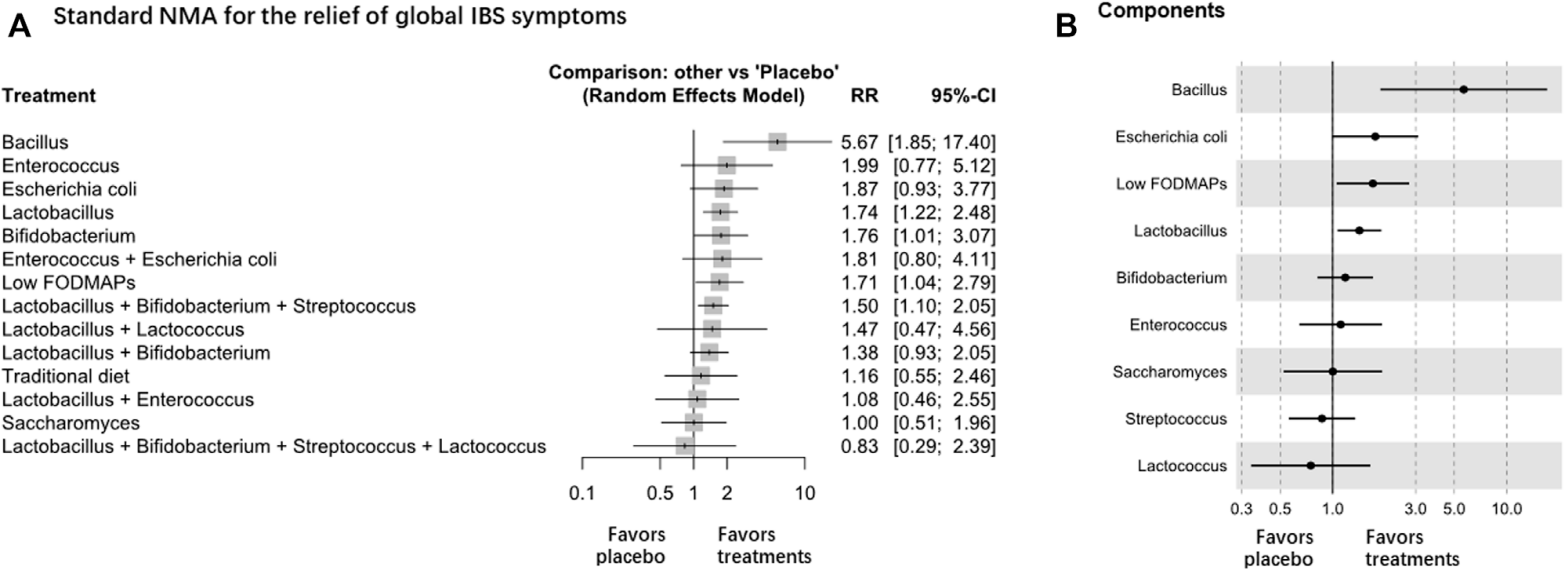
NMA analysis of the relief of global IBS symptoms, **A** is Standard NMA, **B** is Components NMA.

Forty-seven RCTs (n = 5795) were included in the assessment, and *Lactobacillus* and *Bifidobacterium* were among the most effective interventions (high certainty evidence, Table 1 and Figure 2A). The net graphs are shown in Supplementary Figure S2. *Lactobacillus* (RR 1.74, 95% CI 1.22 to 2.48; 8 trials with 932 participants), *Bifidobacterium* (RR 1.76, 95% CI 1.01 to 3.07; 4 trials with 971 participants), and *Bacillus* (Madempudi et al., 2019) (RR 5.67, 95% CI 1.85 to 17.40, 1 trial with 136 participants) were superior to the placebo in improving global IBS symptoms (global *I* ^*2*^ = 71.1%), but no difference was found between *Lactobacillus* and other treatments. *Escherichia coli* (Enck et al., 2009; Kruis et al., 2012) *Saccharomyces*, and *Enterococcus* (Gade and Thorn, 1989) were among the least effective interventions (Table 1). Compared with the placebo, the combination of *Bifidobacterium*, *Lactobacillus*, and *Streptococcus* (RR 1.50, 95% CI 1.10 to 2.05; 9 trials with 892 participants) was the most effective among all the treatment combinations (Table 1 and Figure 2A). Low FODMAP diet (8 trials) and conventional diets (7 trials) could also be among the least effective interventions. Sensitivity analysis showed similar results, and the global *I* ^*2*^ decreased to 40.4%. The estimates were consistent in direct and indirect estimates (Supplementary Figure S3). We also analyzed the RCTs of IBS-D and found that there was no significant difference in the relief of global IBS symptoms among all intervention probiotics (Supplementary Figures S4, S5).

**TABLE 1:** Final classification of 14 interventions, based on network meta-analysis of interventions for IBS (global IBS symptom relief).

Final classification of 11 interventions, based on network meta-analysis of interventions for IBS (global IBS symptom or abdominal pain scores)		
Certainty of the evidence, and classification* of intervention	Intervention†	Interventions vs. placebo (mean difference (95%credible interval))
High certainty (moderate to high certainty evidence)		
Category 1: inferior to the most effective, or superior to the least effective	*Lactobacillus* (M)	-0.90 (-1.54 to -0.27)
Category 0: among the least effective	*Bifidobacterium* (M)	-0.68 (-1.71 to 0.34)
Low certainty (low to very low certainty evidence)		
Category 2: might be among the most effective	*Bacillus* (L)	-2.31 (-3.91 to -0.71)
Category 1: might be inferior to the most effective or superior to the least effective	Low FODMAP diet (L)	-1.46 (-2.64 to -0.29)
	*Bifidobacterium* + *Lactobacillus* + *Streptococcus* (L)	-1.51 (-2.18 to -0.85)
	*Bifidobacterium* + *Lactobacillus* (L)	-1.28 (-2.19 to -0.36)
Category 0: might be among the least effective	*Bacillus* + *Streptococcus* (VL)	-2.24 (-4.53 to 0.06)
	Conventional diet (L)	-1.21 (-2.68 to 0.27)
	*Saccharomyces* (L)	-0.15 (-1.12 to 0.83)
	High FODMAP diet (VL)	3.81 (1.03–6.59)

Abbreviations: IBS, irritable bowel syndrome; FODMAP, fermentable oligosaccharides, disaccharides, monosaccharides, and polyols; H, high certainty evidence; M, moderate certainty evidence; L, low certainty evidence; VL, very low certainty evidence.

Categories do not inform value judgments about the importance of the effects. Letters in brackets represent the certainty of the evidence for each intervention when compared with the reference.

**TABLE 2:** Final classification of 11 interventions, based on network meta-analysis of interventions for IBS (global IBS symptom or abdominal pain scores).

Certainty of the evidence, and classification* of intervention	Intervention†	Interventions vs. placebo (mean difference (95%credible interval))
High certainty (moderate to high certainty evidence)		
Category 1: inferior to the most effective, or superior to the least effective	*Lactobacillus* (M)	-0.90 (-1.54 to -0.27)
Category 0: among the least effective	*Bifidobacterium* (M)	-0.68 (-1.71 to 0.34)
Low certainty (low to very low certainty evidence)		
Category 2: might be among the most effective	*Bacillus* (L)	-2.31 (-3.91 to -0.71)
Category 1: might be inferior to the most effective or superior to the least effective	Low FODMAP diet (L)	-1.46 (-2.64 to -0.29)
	*Bifidobacterium* + *Lactobacillus* + *Streptococcus* (L)	-1.51 (-2.18 to -0.85)
	*Bifidobacterium* + *Lactobacillus* (L)	-1.28 (-2.19 to -0.36)
Category 0: might be among the least effective	*Bacillus* + *Streptococcus* (VL)	-2.24 (-4.53 to 0.06)
	Conventional diet (L)	-1.21 (-2.68 to 0.27)
	*Saccharomyces* (L)	-0.15 (-1.12 to 0.83)
	High FODMAP diet (VL)	3.81 (1.03–6.59)

Abbreviations: IBS, irritable bowel syndrome; FODMAP, fermentable oligosaccharides, disaccharides, monosaccharides, and polyols; H, high certainty evidence; M, moderate certainty evidence; L, low certainty evidence; VL, very low certainty evidence.

Categories do not inform value judgments about the importance of the effects. Letters in brackets represent the certainty of the evidence for each intervention when compared with the reference.

Component NMA showed that *Bacillus* and *Lactobacillus* were among the most effective components to relieve global IBS symptoms (Figure 2B). Component NMA showed that *Bacillus* (Hun, 2009; Rogha et al., 2014; Madempudi et al., 2019; Catinean et al., 2019) (RR 5.67, 95% CI 1.88 to 17.08; *p* = 0.002) and *Lactobacillus* (RR 1.42, 95% CI 1.07 to 1.91; *p* = 0.017) were the most effective components among the treatments.

### Scores of IBS Symptoms or Abdominal Pain

**FIGURE 3:**
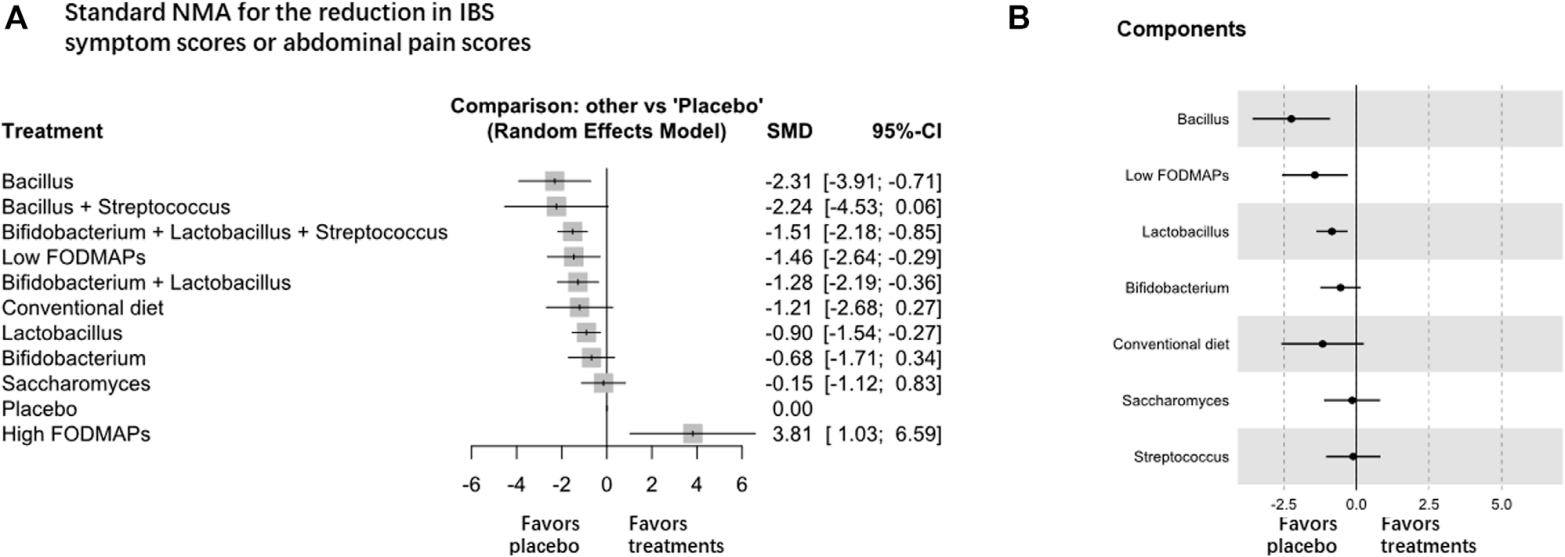
NMA analysis of the reduction in global IBS symptom scores or abdominal pain scores, **A** is Standard NMA, **B** is Components NMA.

For the reduction in IBS symptom scores and abdominal pain scores, forty-five RCTs (n = 5783) were included for analysis, and *Lactobacillus* was the most effective intervention (high certainty evidence, Table 1 and Figure 3A; Supplementary Figure S6). *Lactobacillus* (SMD -0.90, 95% CI −1.54 to −0.27, 10 trials with 1,399 participants), *Bacillus* (SMD –2.31, 95% CI -3.91 to -0.71, 4 trials with 332 participants), and low FODMAP diet (SMD -1.46 95% CI -2.64 to -0.29, 6 trials with 508 participants) were superior to the placebo (global *I* ^*2*^ = 96.2%), but no difference was found among *Lactobacillus*, low FODMAP diet, and other treatments. The combination of *Bifidobacterium*, *Lactobacillus*, and *Streptococcus* (SMD -1.51, 95% CI -2.18 to -0.85, 11 trials with 961 participants), and the combination of *Bifidobacterium* and *Lactobacillus* (SMD -1.28, 95% CI -2.19 to -0.36, 6 trials with 480 participants) also showed significant superiority over placebo. Sensitivity analyses were not performed because the design-by-treatment analysis found that most of the designs contributed to significant heterogeneity. The direct and indirect estimates were consistent (Supplementary Figure S7). No significant difference was observed in the reduction of the global IBS symptom score or abdominal pain score of IBS-D among all intervention probiotics (Supplementary Figures S8, S9).

Component NMA showed that *Bacillus*, low FODMAP diet, and *Lactobacillus* were among the most effective components to improve overall IBS symptom scores or abdominal pain scores (Figure 3B). Component NMA showed that *Bacillus* (SMD -2.09, 95% CI -3.36 to -0.82; *p* = 0.001), *Lactobacillus* (SMD -0.76, 95% CI -1.34 to -0.18; *p* = 0.010), and low FODMAP diet (SMD 1.49 95% CI 0.33 to 2.65; *p* = 0.012) were the most effective components among the treatments.

### Adverse Events

The incidence of adverse events in the probiotics group was higher than that in the low FODMAP diet group. The highest incidence was a gastrointestinal reaction, which could be relieved without special treatment. Twenty-six RCTs reported total adverse events in 3,969 patients. Overall, 315 (16.4%) of the 1921 participants assigned to probiotics had any adverse events, while 230 (14.3%) of the 1,607 participants assigned to the placebo had any adverse events. Five of 108 patients (4.6%) were assigned to a low FODMAP diet, while six of 75 patients (8.0%) were assigned to a placebo. The main adverse reactions caused by probiotics are gastrointestinal symptoms, including abdominal pain, abdominal distention, bloating, flatulence, constipation, diarrhea, vomiting, and nausea. Other events included headache, nausea, urticaria, and bloating, rash, fatigue, itching, ear pain, and cold symptoms. The only adverse reaction associated with a low FODMAP diet was the deterioration of gastrointestinal symptoms. Among all patients taking probiotics, *Bacillus* had the highest incidence of adverse events, with 17 of 23 participants (74.0%). The second was *Enterococcus* + *Escherichia coli*: 52 (149 participants) (34.9%), and the third was *Lactobacillus* + *Enterococcus*: 35 (124 participants) (28.2%).

## Discussion

Our study found that *Lactobacillus*, *Bifidobacterium*, *Bacillus,* and low FODMAP diet are effective components in the management of diet and probiotics to alleviate IBS symptoms. The GRADE evaluation suggests that there is high-quality evidence supporting the effectiveness of *Lactobacillus*. Owing to the limited number of included studies and sample size, the conclusion of *Bacillus* needs further studies. Regarding the treatment combinations, the combination of *Lactobacillus* and *Bifidobacterium* and the combination of *Lactobacillus*, *Bifidobacterium*, and *Streptococcus* are effective, but the conclusion needs to be further verified because of the low certainty of evidence and the small effect size. These treatments have low efficacy while they are not better than the other treatments, and therefore will not help all the patients but more of a subgroup. At the qualitative level, we found that *Lactobacillus*, *Bifidobacterium*, and *Bacillus* were slightly superior to other interventions, but considering the high heterogeneity of the study, this may not be of significant clinical relevance.

We adopted the component NMA method and the GRADE approach for concluding NMA results. Component NMA has advantages in analyzing the effect of complex interventions and identifying the most effective component, compared with the standard NMA(Rücker et al., 2020). Our study showed that the 95% CIs were narrower in component NMA than the standard NMA, indicating a more accurate estimation. In addition, disconnected networks in standard NMA could be solved by component NMA, which therefore added more study power to the NMA analysis. The network of standard NMA was formed through a common comparator, which might not be adopted in most of the studies—for example, the usual care control. However, if the usual care control has a common component—for example, the *Lactobacillus*, the network could be connected in component NMA, which makes the network connection more stable (Rücker et al., 2021). Regarding the minimally contextualized framework recommended by the GRADE group, it facilitates drawing reliable conclusions from NMA (Brignardello-Petersen et al., 2020). Previous NMA relied heavily on treatment rankings, which might vary significantly by including or excluding a single study, and the framework focused mainly on the effect size of treatment, the accuracy of effect estimation, and the RoB of the included RCTs.

The evidence for the effectiveness of probiotics for IBS was more certain than the evidence for a low FODMAP diet, according to the results of our study. The previous meta-analysis confirmed the superiority of probiotics over placebo, but it is unclear which probiotic strains were more effective (Ford et al., 2014a). One NMA comparing the effects of differential probiotics for IBS showed that the combination of *Lactobacillus* and *Bifidobacterium* had a better treatment effect than other probiotic strains (Liang et al., 2019), which was similar to our study result. However, this study included only 14 studies and 1,695 participants; our study had a larger number of studies, further determined which components are more effective, and evaluated the certainty of evidence by using the new GRADE approach.

However, our findings differed from a systematic review (Le Morvan de Sequeira et al., 2021) and the statement on the probiotic treatment of the AGA (Su et al., 2020). AGA is the analysis of a single strain and the study at the genus level or combination; the results make no recommendations for the use of probiotics in children and adults with IBS. The systematic evaluation mainly evaluated the effects of strains on quality of life, anxiety, and depression; it was found at the qualitative level, and probiotic treatment was not superior to placebo. Our study is an analysis of all strains and various combinations, mainly to evaluate the improvement of global symptoms and abdominal pain.

The low FODMAP diet was associated with a larger effect on IBS symptoms compared with other diets, as shown by a recent systematic review (Su et al., 2019), but only one study confirmed that a low FODMAP diet was superior to a placebo or sham diet (Staudacher et al., 2017). The low FODMAP diet is effective to relieve symptoms (Irvine et al., 2016) and improving the quality of life with IBS in comparison with habitual diet or high FODMAP diet (Schumann et al., 2018), and these results were supported by a recent NMA comparing different styles of diet (Black et al., 2021). Dionne et al. found very low-quality evidence to support the recommendation of a low FODMAP diet for IBS patients (Dionne et al., 2018). Our NMA showed inconsistent results in the comparison between low FODMAP diet and placebo; the low FODMAP diet exhibited better effects in the reduction of IBS symptom scores or abdominal pain scores, but not the relief of global IBS symptoms. Further studies are therefore needed to confirm the efficacy of a low FODMAP diet.

Our study demonstrated that *Lactobacillus*, *Bacillus*, *Bifidobacterium*, and low FODMAP diet were effective components in the management methods to the IBS diet and probiotics, and the effectiveness of *Lactobacillus* was the most certain—indicating a recommendation of it in the dietary scheme for patients with IBS. The duration of probiotic administration commonly ranged from 4 to 8 weeks; the optimal treatment duration required is unclear, which warrants future studies especially for probiotics containing *Lactobacillus*. The evidence from low FODMAP diet studies has shown that abdominal bloating or distension severity and bowel habit are the symptoms most improved by the diet (Black et al., 2021); our study did not include these indicators, so we cannot deny the improvement of IBS symptoms by low FODMAP diets.

Our study had several limitations. First, the number of RCTs examining the effects of probiotics and low FODMAP diet was large, and the relevant literature may not have been thoroughly examined. Second, the definitions of patient inclusion criteria are very broad, and there is no unified standard from Manning standard to Rome IV; this heterogeneity clouds the interpretation of data. Several systematic reviews and network meta-analyses on the efficacy of IBS with similar inclusion criteria (Ford et al., 2014b; Ford et al., 2018; Black et al., 2020). Third, the heterogeneity was large in the analysis of the secondary efficacy outcome. The Manning criteria and the 4 variations of Rome describe a rather different type of patient. Abdominal pain was not required for the diagnosis of Rome I and II (Mearin et al., 2004) but for Rome III and Rome IV (Lacy et al., 2016). Fifty-one (67.1%) RCTs were diagnosed with IBS through Rome III and Rome IV, and 25 (32.9) were diagnosed by Rome I, Rome II, and others. The included studies varied according to the age and the proportion of women, of which 67 (88.2%) studies were concentrated in the age range of 30–50 years, and 61 (80.3%) studies had more than half of women. Although we used a random-effects model, the results might have been influenced by imbalanced baseline characteristics and should be interpreted with caution, and to reduce heterogeneity, subgroup analyses were performed. Fourth, we did not assess the comparative cost-effectiveness among the differential probiotics and low FODMAP diets owing to the lack of original studies. The recommendation of specific probiotic strains should be considered together with economic efficiency. Last, our analysis might be underpowered. *Bifidobacterium* showed an RR value of 1.76 (1.01–3.07), while the low FODMAP diet showed nearly identical values of 1.71 (1.04–2.79). The results showed low FODMAP diet showed a similar effect size as *Bifidobacterium*, but it was classified as an insignificant difference. The results indicated that we should judge the clinical relevance before we apply the findings of this study into practice, and it also indicated a necessity for more research in this field.

In conclusion, we found that *Lactobacillus*, *Bifidobacterium*, *Bacillus*, and low FODMAP diet were effective in alleviating global IBS symptoms, suggesting that there is high-quality evidence supporting the effectiveness of *Lactobacillus*. The GRADE approach suggests that there is high-quality evidence supporting the effectiveness of *Lactobacillus.* The effectiveness of other components should be further examined in future studies.

## Key Points

• Low FODMAP diet and probiotics are two commonly adopted interventions for patients with IBS before the initiation of pharmacological treatments. The most effective component in these interventions has not been clarified.
• With high certainty evidence, *Lactobacillus* was among the most effective components for the relief of global IBS symptoms.
• *Bifidobacterium* and *Bacillus* were possibly effective and should be further verified.

## Data Availability

The original contributions presented in the study are included in the article/Supplementary Material, further inquiries can be directed to the corresponding authors.
