# Supplementary material for: Low FODMAP Diet and Probiotics in Irritable Bowel Syndrome: A Systematic Review With Network Meta-analysis
Source: Front Pharmacol. 2022 Mar 9;13:853011. doi: 10.3389/fphar.2022.853011 (PMC8959572; doi:10.3389/fphar.2022.853011)
Supplement: Supplementary file 1 [file Table1.pdf]

Supplementary Table 1. Study characteristics

Author (year)	Design	Sample size (% female, mean age)	Participants	Interventions	Outcomes
1. Combination					
Kim, (2003)	USA RCT, Single-center	25 (72,42.8)	Rome II, 100% IBS-D	Combination VSL#3 (225 billion bacteria/packet) bid for 8 weeks	SR of IBS symptoms for 50% of weeks
Kim, (2005)	USA RCT, Single-center	48 (93.8,43)	Rome II, 42 % IBS-D, 33% IBS-C, 25% IBS-M	Combination VSL#3 bid (31 patients for 4 weeks and 17 patients for 8 weeks)	SR of IBS symptoms for 50% of weeks
Kajander, (2005)	Finland RCT, Single-center	103 (76.5,45.5)	Rome I and II, 47 .6% IBS-D, 23.3% IBS-C, 29.1% IBS-M	Combination *LGG*, *LR* LC705, *Propionibacterium Free-denreichii*, and *B. breve* Bb99 (8-9×10^9 cfu/capsule) for 6 months	Relief of global symptom score
Kim, (2006)	Korea RCT, Single-center	40 (26.5,39.4)	Rome II, 70 % IBS-D, 30% IBS-M	Combination *Bacillus subtilis* (1×10^9), *S. faecium* (9×10^9) tid for 4 weeks.	Abdominal pain
Guyonnet, (2007)	France RCT, Thirty-five-center	274 (74.5,49.3)	Rome II, 100% IBS-C	Combination *B. animalis* DN 173010 (1.25×10^{10} cfu/125g), *ST* (1.2×10^9 cfu/125g) and *L. bulgaricus* (1.2×10^9 cfu/125g) bid for 6 weeks	Improvement at least 10% discomfort dimension score, adverse events
Zeng, (2008)	China RRCT, Single-center	30 (34.5,45.2)	Rome II, 100% IBS-D	Combination *ST* (1×10^8 cfu/ml), *L. bulgaricus* (1×10^7 cfu/ml), *LA'* (1×10^7 cfu/ml), and *BL* (1×10^7 cfu/ml) 200ml bid for 4 weeks	Global IBS scores in GSRS
Kajander, (2008)	Finland RCT, Single- center	86 (93,48)	Rome II, 45% IBS-D, 25% IBS-C, 30% IBS-M	Combination *LGG* ATCC 53103, *LGG*, *LR* Lc705 DSM 7061, *P. freudenreichii ssp. Shermanii JS* DSM 7067 and *B. animalis ssp. lactis* Bb12 DSM 15954 (1×10^7 cfu/ml) 1.2dl qd for 14 weeks	Global IBS symptoms score, adverse events
Enck, (2008)	Germany RCT, Ten-center	297 (73.5,49.6)	Clinical criteria, IBS-D, IBS-C, IBS-M	Combination *Enterococcus faecalis* DSM 16440 and *Escherichia coli* DSM 17252 (3.0-9.0×10^7 cfu/1.5ml) ×0.75ml tid for 1 week, then 1.5 ml tid for weeks 2 and 3, then 2.25 ml tid for weeks 3–8	Have at least a 50% decrease in global symptom score, adverse events
Drouault-Holowacz, (2008)	France RCT, Single- center	106 (76,45.4)	Rome II, 29% IBS-D, 29% IBS-C, 41% IBS-M, 1% IBS-U	Combination *BL LA'* 101 (29%), *Lb. acidophilus LA'* 102 (29%), *L. lactis LA'* 103 (29%) and *S. thermo-philus LA'* 104 (13%) 1×10^{10} cfu for 4 weeks	SR of overall IBS symptoms
Williams, (2009)	UK RCT, Single-center	56 (86.5,39)	Rome II, 11.5% IBS-D, 27% IBS-C, 61.5% IBS-M	Combination *LA'* CUL-60 NCIMB 30157 and CUL-21 NCIMB 30156, *BB* CUL-20 NCIMB 30153, and *BL'* CUL-34 NCIMB 30172 (2.5×10^{10} cfu/capsule) for 8 weeks	IBS-SSS
Hong, (2009)	Korea RCT, Single-center	70 (32.9,37)	Rome III, 45.7% IBS-D, 20% IBS-C, 8.5% IBS-M, 25.8% IBS-U	Combination *BB* BGN4, BL' AD011, *L. acidophil* AD031, and *LC* IBS041 (20 Billi bacteria/sachet) bid for 8 weeks	Reduction of symptom score by at least 50%, adverse events
Simren,	Sweden RCT,	74	Rome II,	Combination	AR of IBS symptoms

(2010)	Single-cente	(70.3,43)	35.1% IBS-D, 14.9% IBS-C, 50% IBS-M	*LP ssp paracasei* F1 P9, *LA'*5, and *BL'* Bb 12 200ml (5×10^{7} cfu/ml) bid for 8 weeks	at least 50%
Sondergaard, (2011)	Sweden RCT, Two-center	64 (75,51.2)	Rome II, Subtype not reported	Combination *LP ssp paracasei* F19, *LA'*5 and *BL'* Bb12 (5 x 10^{7} cfu/ml) for 8 weeks	AR of IBS symptoms
Michail, (2011)	USA RCT, Single- center	24 (66.7,21.8)	Rome III, 100% IBS-D	Combination VSL#3 (900 billion bacteria/packet) qd for 8 weeks	Global symptoms score (a clinical rating scale GSRS)
Cui, (2012)	China RCT, Single-center	60 (70,44.7)	Rome III, 48.3% IBS-D, 30% IBS-C, 11.7% IBS-M, 10% IBS-U	Combination *BL* and *LA'* tid for 4 weeks	Improvement in IBS symptoms
Cha, (2012)	Korea RCT, Single-center	50 (48,39.7)	Rome III, 100% IBS-D	Combination *LA'*, *LP'*, *LR*, *B. breve*, *BL', BL*, and *ST* (0.5×10^{10} cfu/capsule) bid for 8 weeks	AR of IBS symptoms at least 50% of the weeks
Ko, (2013)	Korea RCT, Single-center	26 (63.3,37.3)	Rome III, 100% IBS-D	Combination *B. brevis*, *BL'*, and *BL*, *LA'*, *LP'*, and *LR*, and *ST* (5 billion bacteria/capsule) tid for 8 weeks	AR of overall IBS symptoms
Begtrup, (2013)	Denmark RCT, Single-center	131 (30,30.5)	Rome III, 40.5% IBS-D, 19.1% IBS-C, 38.2% IBS-M, 2.2% IBS-U	Combination *LP ssp paracasei* F19, *LA'*5, and *BL'* Bb 124 capsules (1.3×10^{10} cfu/capsule) for 6 months	AR of global IBS symptoms
Roberts, (2013)	UK RCT, Thirteen-center	179 (85,44.2)	Rome III, 100% IBS-C or IBS-M	Combination *BL'* I-2494 (previously known as DN1 73010) (1.25×10^{10} cfu/pot), ST I-1630 (1.2×10^{9} cfu/pot), and *L. bulgaricus* I-1632 and I-1519 (1.2×10^{9} cfu/pot) bid for 12 weeks	Subjective global assessment of symptom relief
Sisson, (2014)	UK RCT, Single-center	186 (69.4,38.3)	Rome III, 37 .6% IBS-D, 21.5% IBS-C, 35.5% IBS-M, 5.4% IBS-U	Combination *LR* NCIMB 30174, *LP'* NCIMB 30173, *LA'* NCIMB 30175 and *Enterococcus faecium* NCIMB 30176 (1×10^{10} cfu/50ml) for 12 weeks	IBS-SSS, adverse events
Ludidi, (2014)	Netherlands RCT, Single-center	40 (67.5,40.5)	Rome III, 42.5% IBS-D, 10% IBS-C, 30% IBS-M, 17 .5% IBS-U	Combination *BL'* W52, LC W56, *L. salivarius* W57, *L.* W58, *LA'* NCFM, and *LR* W71 (5×10^{9} cfu/sachet) for 6 weeks	Mean symptom composite score
Lorenzo-Zuniga, (2014)	Spain RCT, Two-center	84 (63.1,46.8)	Rome III, 100% IBS-D	Combination *LP'* CECT7484 and CECT7485 and one *Pediococcus acidilactici* CECT7483 (high dose:1-3×10^{10} cfu/capsule or high dose: 3-6×10^{9} cfu/capsule qd for 6 weeks	Relief of IBS symptoms
Jafari, (2014)	Iran RCT, Single-center	108 (60.2,36.7)	Rome III, Subtype not reported	Combination *B. animalis subsp. lactis* BB-12®, LA-5®, *L. delbrueckii subsp. bulgaricus* LBY-27, ST STY-31 (4×10^{8} cfu/capsule) bid for 4 weeks	Relief of IBS symptoms
Yoon, (2014)	Korea RCT, Single-center	49 (65.3,44.5)	Rome III, 53.1% IBS-D, 40.8% IBS-C, 6.1% IBS-M	Combination *BB* KCTC 12199BP, *BL'* KCTC 11904BP, *BL* KCTC 12200BP, *LA'* KCTC 11906BP, *LR* KCTC 12202BP and *ST* KCTC 11870BP. (5 x 10^{9} viable cells/capsule) bid for 4 weeks	Global relief of IBS Symptoms
Yoon,	Korea RCT,	81	Rome III,	Combination	AR of overall IBS

(2015)	Single-center	(46.3,59.3)	48.1% IBS-D, 18.5% IBS-C, 21%IBS-M, 12.4%IBS-U	*BB* KCTC 12199BP, *BL'* KCTC 11904BP, *BL* KCTC 12200BP, *LA'* KCTC 11906BP, *LR* KCTC 12202BP, and *ST* KCTC 11870BP (5×10^9 viable cells/capsule) bid for 4 weeks	symptoms
Wong, (2015)	Singapore RCT, Single-center	42 (45.2,47)	Rome III, IBS-M	Combination VSL#3 (225 billion bacteria/capsule) bid for 6 weeks	Overall IBS symptom scores
Hod, (2017)	Israel RCT, Single-center	107 (100,29.5)	Rome III, 100% IBS-D	Combination *LR5* (3×10^9 cfu/capsule), *LC5* (2×10^9 cfu/capsule), *LPC5* (1×10^9 cfu/capsule), *LP'3* (1×10^9 cfu /capsule), *LA'1* (5×10^9 cfu/capsule), *B. bifidum* BF3 (4×10^9 cfu/capsule), *BL* BG7 (1×10^9 cfu /capsule), *B. breve* BR3 (2×10^9 cfu /capsule), *BI* BT1 (1×10^9 cfu/capsule), *ST*3 (2×10^9 cfu/capsule), *L. bulgaricus* LG1, and *Lactococcus lactis* SL6 (3×10^9 cfu/capsule) bid for 8 weeks	Abdominal pain, overall responder rates
Kim, (2020)	Korea RCT, Single-center	63 (74.6,36)	Rome II, 100% IBS-D	Combination *BL* BORI, *BB* BGN4, *BL'* AD011, *BI* IBS007, and *LA'* AD031 (5×10^9 viable cells/capsule) tid for 8 weeks	Relief of IBS symptoms
Barraza-Ortiz, (2020)	México RCT, Single-center	36 (61.5,45.6)	Rome IV, 55.5% IBS-D, 44.5%IBS-M	Combination *LP'* CECT 7484, *LP'* CECT 7485 and *Pediococcus acidilactici* CECT 7483 (3×10^9 cfu/capsule) once daily for 6 weeks	Relief of abdominal pain
Skrzydło-Radomańsk, (2021)	Poland RCT, Single-center	51 (64.5,43.1)	Rome III, 100% IBS-D	Combination *Bifidobacterium breve, BL, BB, BL', LR, LP, LA', LC, LP',* and *ST* (2.08×10^8 cfu/capsule) po bid for 8weeks	IBS-SSS
2. *Lactobacillus*					
Nobaek, (2000)	Sweden RCT, Single-center	60 (69.2,48.5)	Rome criteria, IBS-D, IBS-C	*Lactobacillus* *LP'* DSM 9843 (5×10^7 cfu/ml/drink), 400ml qd for 4 weeks	Significant improvements in the IBS symptoms
Niedzielin, (2001)	Poland RCT, Single- center	40 (80,43.5)	Clinical diagnosis, 2.5% IBS-D, 52.5% IBS-C, 45% IBS-M	*Lactobacillus* *LP'*299V (5×10^7 cfu/ml) bid for 4 weeks	Improvement in global IBS
Niv, (2005)	Israel RCT, Two-center	54 (66.7,45.7)	Rome II, 37% IBS-D, 18.5% IBS-C, 44.4% IBS-M	*Lactobacillus* *L. reuteri* ATCC 55730 (1×10^8 colony-forming units/table), qd for 1 week, then bid a week	Global symptoms score, adverse events
Sinn, (2008)	Korea RCT, Single-center	40 (65,44.7)	Rome III, 10% IBS-D, 27 .5% IBS-C, 62.5% IBS-M	*Lactobacillus* *LA'* SDC 2012 and 2013 (2×10^9 cfu/ml) bid for 4 weeks	Reduction in abdominal pain score
Farup, (2012)	Norway RCT, Single-center	16 (69,50)	Rome II, 37.5% IBS-D, 6.25% IBS-C, 56.25%IBS-M	*Lactobacillus* *LP'*MF 1298 (1×10^{10} cfu/capsule) qd for 6 weeks	Global symptoms score
Ducrotte, (2012)	France RCT, Four-center	214 (29.4,37.3)	Rome III, 62% IBS-D, 38% non-classified	*Lactobacillus* *LP'*299V DSM 9843 (10 billion cfu/capsule) for 4 weeks	Relief of IBS symptoms, adverse events
Dapoigny,	France RCT,	52	Rome III,	*Lactobacillus*	IBS severity score

(2012)	Four-center	(70,47.1)	30% IBS-D, 22% IBS-C, 34% IBS-M, 14% IBS-U	*LCR*35 (2×10^8 cfu/capsule) 250mg tid for 4 weeks	reduced by at least 50%
Murakami, (2012)	Japan RCT, Single- center	35 (56.5,16.2)	Rome III, Subtype not reported	*Lactobacillus* *L. brevis* KB290 ($\geq1.0\times10^{10}$ cfu/capsule) 1 capsule/day for 4 weeks	IBS symptoms scores
Pedersen, (2014)	Denmark RCT, Single- center	123 (73.2,37.3)	Rome III, 40.7% IBS-D, 15.4% IBS-C 38.2% IBS-M	*Lactobacillus*, Low FODMAPs diet *LGG* or Low FODMAPs diet bid for 6 weeks	Reduction of IBS-SSS
Stevenson, (2014)	South Africa RCT, Single-center	81 (97.5,47.9)	Rome III, 37.6% IBS-D, 21.5% IBS-C	*Lactobacillus* *LP'* 299v (5×10^9 cfu/capsule) for 8weeks	IBS-SSS, adverse events
Thijssen, (2016)	Holland RCT, Four-center	80 (68.8,41.8)	Rome II, 30% IBS-D, 25% IBS-C, 28.75% IBS-M, 16.25% IBS-U	*Lactobacillus* *LcS* (6.5×10^9 cfu/bottle) bid for for 8 weeks	A MSS decrease of at least 30%
Lyra, (2016)	Finland RCT, Two-center	391 (74.7,47.9)	Rome III, 38.9% IBS-D, 16.6% IBS-C, 44% IBS-M, 0.5% IBS-U	*Lactobacillus* *LA'* NCFM (ATCC 700396) (low dose: 1×10^9 cfu/capsule, high dose: 1×10^{10} cfu/capsule) for 12 weeks	IBS-SSS, adverse events
Shin, (2018)	Korea RCT, Single- center	60 (56.9,36.5)	Rome III, 100% IBS-D,	*Lactobacillus* *L. gasseri* BNR17 (1×10^{10} cfu/day) bid for 8 weeks	Relief of IBS symptoms
Oh, (2019)	Korea RCT, Single-center	55 (72,32.8)	Rome III, 42% IBS-D, 20% IBS-M, 38% IBS-U	*Lactobacillus* *LP*, *L. salivarius*, and *LP'*, (1×109 cfu/mL) qd for 4 weeks	Relief of IBS Symptoms
Sadrin, (2020)	France RCT, Multi-center	80 (71,48.9)	Rome III, Subtype not reported	*Lactobacillus* *LA'* NCFM and *LA'* subsp. Helveticus *LA'* FTIL 10 (5×10^9 cfu/capsule) bid for 8 weeks	Relief of IBS symptoms score, adverse events
Lewis, (2020)	Canada RCT, Single-center	285 (77.7,42)	Rome III, 15.1% IBS-D, 11.2% IBS-C, 73.7%IBS-M	*Lactobacillus/ Bifidobacterium* *LP* HA-196 or *BL* R0175 (10×10^9 cfu/capsule) qd for 8 weeks.	IBS-SSS, adverse events
Martoni, (2020)	USA RCT, Twelve-center	336 (49.5,39.5)	Rome III, Subtype not reported	*Lactobacillus /Bifidobacterium* *LA'* DDS-1 or *BL'* UABla-12 (1×10^{10} cfu/capsule) qd for 6 weeks	Relief of IBS symptoms score
3. Low FODMAPs diet					
Staudacher, (2012)	UK RCT, Single-center	41 (65.8,35.5)	Rome III, 100% IBS-D	Low FODMAPs diet Low FODMAPs diet for 4 weeks	Adequate control of IBS symptoms, adverse events
Pedersen, (2014)	Denmark RCT, Single- center	123 (73.2,37.3)	Rome III, 40.7% IBS-D, 15.4% IBS-C 38.2% IBS-M	Low FODMAPs diet, *Lactobacillus* Low FODMAPs diet or *LGG* bid for 6 weeks	Reduction of IBS-SSS
Böhn, (2015)	Sweden RCT, Three- center	75 (81.3,42.5)	Rome III, 24% IBS-D, 29.3% IBS-C, 46.7%IBS-M/U	Low FODMAPs diet Low FODMAPs diet for 4 weeks	Reduction in IBS severity scores ≥50
Chumpitazi, (2015)	USA RCT, Single- center	33 (67,11.5)	Rome III, 9.1% IBS-D, 72.7% IBS-C, 9.1% IBS-M, 9.1% IBS-U	Low FODMAPs diet Low FODMAPs diet for 2 weeks	Abdominal pain frequency
Eswaran, (2016)	US RCT, Single-center	92 (70.7,41.7)	Rome III, 100% IBS-D	Low FODMAPs diet Low FODMAPs diet for 4 weeks	AR of IBS-D symptoms ≥50%
McIntosh, (2017)	Canadian RCT, Single- center	40 (86.4,50.9)	Rome III, 25% IBS-D, 5% IBS-C,	Low FODMAPs diet Low FODMAPs diet for 3 weeks	IBS symptom reduction ≥50

			57.5% IBS-M, 2.5% IBS-U		
Staudacher, (2017)	UK RCT, Two- center	104 (67.5,35.5)	Rome III, 66.3% IBS-D, 23.1% IBS-M, 10.6% IBS-U	Low FODMAPs diet Low FODMAPs diet/ placebo or low FODMAPs diet/ probiotic (VSL#3) for 4 weeks	AR of IBS symptoms, adverse events
Patcharatrakul, (2019)	Thailand RCT, Single-center	66 (75.8,51)	Rome III, 100% IBS-C	Low FODMAPs diet Structural individual low FODMAPs dietary advice for 4 weeks	Relief of abdominal pain
Darvishmoghadam, (2019)	Iran RCT, Single-center	50 (58,30.1)	Rome IV, 100% IBS-D	Low FODMAPs diet Low FODMAPs diet (almond) 40g/day for 20 days	Relief of IBS symptoms
Wilson, (2020)	UK RCT, Single-center	69 (55.2,34.1)	Rome III, 65.3% IBS-D, 34.7% IBS-U	Low FODMAPs diet with placebo for 4 weeks.	Global symptom question
Goyal, (2021)	India RCT, Single-center	101 (58.1,42)	Rome IV, Subtype not reported	Low FODMAPs diet Low FODMAPs diet (almond) for 4 weeks	Reduction of IBS-SSS, reduction in IBS-SSS ≥ 50
4. *Bifidobacterium*					
Whorwell, (2006)	UK RCT, Twenty-center	362 (100,41.9)	Rome II, 55.5% IBS-D, 20.7% IBS-C, 23.8% IBS-M,	*Bifidobacterium* *BI* 35624 (1×10^6 live bacteria/capsule, 1×10^8 live bacteria/capsule, or 1×10^{10} live bacteria/capsule) for 4 weeks	Relief of overall IBS symptoms
Agrawal, (2009)	UK RCT, Single-center	38 (100,39.5)	Rome III, 100% IBS-C	*Bifidobacterium* *BL'* DN 173010 1.25×10^{10} cfu/pot [*ST* and *L. bulgaricus* (1.2×10^9 cfu/pot)] qd for 4 weeks	Global symptoms score
Guglielmetti, (2011)	Germany RCT, Multi-center	122 (67.2,38.9)	Rome III, 21.3% IBS-D, 19.7% IBS-C, 59% IBS-M	*Bifidobacterium* *BB* MIMBb75 (1×10^9 cfu/capsule) qd for 4 weeks	Relief of overall IBS symptoms, adverse events
Charbonneau, (2013)	USA RCT, Single-center	76 (81.7,45.1)	Rome II, Subtype not reported	*Bifidobacterium* *BI* 35624 (109 cfu per capsule, range 10^8–10^{10} cfu/capsule) qd for 4 weeks	The severity of gastrointestinal symptoms
Pinto-Sanchez, (2017)	Canada RCT, Single- center	44 (54,43.3)	Rome III, 61.4% IBS-D, 38.6% IBS-M	*Bifidobacterium* *BL* NCC3001 *subspecies longum* (1.0E + 10cfu /1g ram powder with maltodextrin) for 6 weeks.	AR of IBS symptoms
Andresen, (2020)	Germany RCT, Twenty-center	443 (69.5,41.4)	Rome III, 40% IBS-D, 24.1% IBS-C, 7.7% IBS-M, 28.2% IBS-U	*Bifidobacterium* *BB* HIMIMBb75 (1×10^9 cells/capsule) qd for 8 weeks	AR of IBS symptoms, adverse events
Lewis, (2020)	Canada RCT, Single-center	285 (77.7,42)	Rome III, 15.1% IBS-D, 11.2% IBS-C, 73.7%IBS-M	*Lactobacillus/ Bifidobacterium* *LP* HA-196 or *BL* R0175 (10×10^9 cfu/capsule) qd for 8 weeks.	IBS-SSS, adverse events
Martoni, (2020)	USA RCT, Twelve-center	336 (49.5,39.5)	Rome III, Subtype not reported	*Lactobacillus /Bifidobacterium* *LA'* DDS-1 or *BL'* UABla-12 (1×10^{10} cfu/capsule) qd for 6 weeks	Relief of IBS symptoms score
5. *Saccharomyces*					
Choi, (2011)	Korea RCT, Three-center	90 (46,40.4)	Rome II, 71.6 % IBS-D, 28.4% IBS-M	*Saccharomyces* *Saccharomyces boulardii* (2×10^{11} live cells/capsule) bid for 4 weeks	Overall improvement in IBS-QOL, adverse events
Abbas, (2014)	Pakistan RCT, Single-center	72 (26.4,35.4)	Rome II, 100 % IBS-D	*Saccharomyces* *Saccharomyces boulardii* 750 mg/day for 6 weeks (week 3–week 8).	Abdominal pain, adverse events
Pineton de Chambrun, (2015)	France RCT, Single- center	200 (86,44)	Rome III, 28.5% IBS-D, 46.9% IBS-C, 24.6% IBS-M	*Saccharomyces* *Saccharomyces cerevisiae* CNCMI-3856 (4×10^9 cfu/capsule) for 8 weeks	Improvement of abdominal pain, adverse events

Spiller, (2016)	UK RCT, Single- center	379 (83.6,45.4)	Rome III, 20.8 % IBS-D, 47.5 % IBS-C, 31.7% IBS-M	*Saccharomyces* *Saccharomyces cerevisiae* I-3856 (1000mg, 8×10^9 colony forming units cfu/g) qd for 12 weeks	Improvement of 50% of the weekly average "intestinal pain/discomfort score", adverse events
Gayathri, (2020)	India RCT, Single-center	100 (34,41)	Rome III, 65 % IBS-D, 24 % IBS-C, 11% IBS-M	*Saccharomyces* Standard treatment for 2 weeks then *Saccharomyces cerevisiae* CNCM I-3856 (2×10^9 cfu/capsule) bid for 8 weeks	Abdominal pain score, adverse events
6. *Bacillus*					
Hun, (2009)	USA RCT, Single-center	50 (82.0,48.0)	Rome II, 100 % IBS-D	*Bacillus* *Bacillus coagulans* GBI-30, 6086 qd for 8 weeks	The severity of IBS symptoms
Rogha, (2014)	Iran RCT, Single-center	85 (78.6,39.8)	Rome III, 32 % IBS-D, 12.5 % IBS-C, 50% IBS-M, 5.4% IBS-U	*Bacillus* *Bacillus Coagulans* (15×10^7 Spores) and Fructo-oligosaccharides (100 mg) tid for 12 consecutive weeks	The severity of gastrointestinal symptoms
Catinean, (2019)	Romania RCT, Single-center	90 (60,39.4)	Rome III, 100 % IBS-D	*Bacillus* *Bacillus spp* qd for 1 week then bid for 24 days	IBS-SSS
Madempudi, (2020)	India RCT, Single-center	136 (27.8,43.4)	Rome III, IBS-D, IBS-C, IBS-M, IBS-U	*Bacillus* *Bacillus coagulant Unique* IS2 (2 billion cfu/capsule) qd for 8 weeks	Relief of abdominal pain, satisfactory relief of IBS symptoms
7. Another					
Enck, (2009)	Germany RCT, Twelve-center	298 (49.3,49.6)	Others, IBS-D, IBS-C, IBS-M	*Escherichia coli* *E. coli* DSM1 7252 ($1.5\text{-}4.5\times10^7$ cfu/ml) 0.75ml drops tid for 1 week, then 1.5ml tid for weeks 2-8	AR of IBS core symptoms, adverse events
Kruis, (2012)	Germany RCT, Single-center	120 (76.7,45.7)	Rome II, 45% IBS-D, 29.2% IBS-C, 25.8%IBS-M/U	*Escherichia coli* *E. coli Nissle* 1917 ($2.5\text{-}25\times10^9$ cfu/capsule) for 4 days then bid for 12 weeks	Clinical response (patients reported satisfied in treatment), adverse events
Gade, (1989)	Denmark RCT, Thirteen-center	54 (77.8,34)	Others, IBS-D, IBS-C	*Enterococcus* *S. faecium*, 4 tablets bid for 4 weeks	Improvement in IBS symptoms

Abbreviations: RCT, randomized controlled trial. IBS, irritable bowel syndrome. IBS-C, constipation-predominant irritable bowel syndrome. IBS-D, diarrhea-predominant irritable bowel syndrome. IBS-M, mixed irritable bowel syndrome. IBS-U, un-subtyped irritable bowel syndrome. FODMAPs, fermentable oligosaccharides, disaccharides, monosaccharides, and polyols. IBS-SSS, irritable bowel syndrome symptom severity score. IBS-QOL, evaluation of the irritable bowel syndrome quality of life. GSRS, gastrointestinal symptom rating scale. MSS, mean symptom score. SR, satisfactory relief. AR, adequate relief. USA, United States of America. UK, United Kingdom. *L*, *Lactobacillus*. *B*, *Bifidobacterium*. *S*, *Streptococcus*. VSL#3, *Bifidobacterium longum* (*BL*), *Bifidobacterium infantis* (*BI*), *Bifidobacterium breve*, *Lactobacillus acidophilus* (*LA'*), *Lactobacillus casei* (*LC*), *Lactobacillus delbrueckii ssp*, *Bulgaricus*, *Lactobacillus plantarum* (*LP'*), *Streptococcus salivarius ssp*, *thermophiles*. *LcS*, *Lactobacillus casei Shirota*. *LGG*, *Lactobacillus rhamnosus GG*. *LP*, *Lactobacillus paracasei*. *LR*, *Lactobacillus rhamnosus*. *BB*, *Bifidobacterium bifidum*. *BL'*, *Bifidobacterium lactis*. *ST*, *Streptococcus thermophilus*.
